# OVATE Family Protein PpOFP1 Physically Interacts With PpZFHD1 and Confers Salt Tolerance to Tomato and Yeast

**DOI:** 10.3389/fpls.2021.759955

**Published:** 2021-11-12

**Authors:** Qiuping Tan, Shan Jiang, Ning Wang, Xiao Liu, Xinhao Zhang, Binbin Wen, Yuhui Fang, Huajie He, Xiude Chen, Xiling Fu, Dongmei Li, Wei Xiao, Ling Li

**Affiliations:** ^1^College of Horticulture Science and Engineering, Shandong Agricultural University, Tai’an, China; ^2^State Key Laboratory of Crop Biology, Shandong Agricultural University, Tai’an, China; ^3^Shandong Collaborative Innovation Center for Fruit and Vegetable Production With High Quality and Efficiency, Tai’an, China; ^4^College of Life Sciences, Shandong Agricultural University, Tai’an, China; ^5^Shandong Huayu University of Technology, Dezhou, China

**Keywords:** *Prunus persica*, salt tolerance, OVATE Family Protein PpOFP1, ZF-HD_dimer domain protein PpZFHD1, protein interaction

## Abstract

The OVATE family protein (OFP) genes (*OFPs*) have been shown to respond to salt stress in plants. However, the regulatory mechanism for salt tolerance of the peach (*Prunus persica*) OFP gene *PpOFP1* has not been elucidated. In this study, using yeast two-hybrid screening, we isolated a nucleus-localized ZF-HD_dimer domain protein PpZFHD1, which interacts with the PpOFP1 protein in the peach cultivar “Zhongnongpan No.10”. A segmentation experiment further suggested that the interaction happens more specifically between the N-terminal, contains ZF-HD_dimer domain, of PpZFHD1 and the C-terminal, consists of OVATE domain, of PpOFP1. Additionally, quantitative real-time polymerase chain reaction (qRT-PCR) experiments indicate that transcription of these two genes are induced by 200 mmol/L (mM) NaCl treatment. Heterogeneous transformation experiments suggested that the growth status of transformed yeast strain over-expressing each of these two genes was more robust than that of control (CK). Furthermore, transgenic tomato plants over-expressing *PpOFP1* were also more robust. They had a higher content of chlorophyll, soluble proteins, soluble sugars, and proline. Activities of the superoxide dismutase (SOD), peroxidase (POD), and catalase (CAT) in these plants were higher, and tissues from these plants exhibited a lower relative conductivity and malondialdehyde (MDA) content. These results suggest that PpOFP1 physically interacts with PpZFHD1 and confers salt tolerance to tomato and yeast, thus revealing a novel mechanism for regulating salt tolerance in peach and other perennial deciduous trees.

## Introduction

### Research Progress of Fruit Trees in Response to Salt Stress

The extremity of agricultural salt damage has increased significantly in the 21st century. The extent of salinization has approached 900 million hm^2^ globally, and includes some 100 million hm^2^ in China alone (Fang et al., 2021). The effects of salt damage on fruit trees vary with climate conditions, light intensity, soil conditions, and plant species. Osmotic regulation, chlorophyll content, antioxidant defense system, and cell membrane stability are all affected by salt stress (Singh and Sharma, 2018). Under salt stress the chlorophyll content in grape leaves decreased differentially among varieties (Sivritepe and Eriş, 1999). Yuan et al. (2016) found that VaPAT1, a transcription factor of the GRAS family from *Vitis amurensis*, can respond to salt stress. Furthermore, over-expressing VaPAT1 in Arabidopsis increase the salt tolerance of transgenic lines by increasing the proline and soluble sugar content. Transgenic lemon plants over-expressing FcWRKY40 exhibit increased salt tolerance, increased proline content, and improved ion transport (Dai et al., 2018). When stressed with salt, the soluble sugar content of papaya varieties with strong salt tolerance increased. In salt sensitive varieties it initially increased, but decreased over time (Chander and Palaniappan, 2016). Al-Shorafa et al. (2014) reported that after salt treatment proline accumulated in strawberry leaves and membrane permeability increased.

### Plant OVATE Family Protein Transcription Factors

The OFP transcription factor containing an OVATE domain composing of 70 amino acids, was first identified in pear-shaped tomato (Liu et al., 2002). Zhang et al. (2020) found that AtOFP2 and AtOFP5 could interact directly with the tubule regulatory protein TONNEU2 (TON2), and alter the normal development and cell morphology of the embryo sac. An interaction of AtOFP4, AtKNATs, and AtBLHs proteins in Arabidopsis regulates the formation of secondary cell walls (Liu and Douglas, 2015). The interaction between AtOFP1 and AtKu of Arabidopsis can regulate the DNA repair process. In rice, OsOFP8 protein can interact with OsGSK2 and be phosphorylated, which helps to regulate the feedback circuit of BR signaling, thus affecting hormone response in tissues (Yang et al., 2016). CaOvate inhibited the expression of *CaGA20ox1* gene, thus changing the shape of pepper fruit (Tsaballa et al., 2011). The co-expression of MaMADS1 and MaOFP1 proteins in banana can alter fruit shape and improve quality related to hardness, soluble solid, and soluble sugar content (Liu et al., 2017).

In addition to the regulation of plant growth and development, the *OFPs* gene play key roles in the process of coping with abiotic stress. Drought stress can induce the expression of *AtOFP8*, and the drought resistance of Arabidopsis overexpressing the *AtOFP8* gene is enhanced (Tang et al., 2018). Rice plants over-expressing *OsOFP6* showed stronger drought and cold resistance, while the inhibition of *OsOFP6* expression resulted in hypersensitivity, indicating that it played an important role in coping with abiotic stress (Ma et al., 2017). It is suggested that *MdOFP*s in apple may participate in the regulation of salt stress responses (Li et al., 2019).

### Zinc Finger Homologous Domain Proteins in Plants

Zinc finger homologous domain proteins are plant-specific transcription factors which were first identified in the C4 plant chrysanthemum (Windhövel et al., 2001). They have been implicated in the regulation of the gene coding process of PEPCase (Windhövel et al., 2001). They can specifically identify and bind nucleic acids or proteins (Takatsuji, 1999; Krishna et al., 2003; Wang et al., 2016), regulate gene expression, and play an important role in stress response and defense activation of plants (Mackay and Crossley, 1998). Many studies have identified ZF-HD gene families in Arabidopsis, peach, apple, land cotton, and coconut (Xu et al., 2014; Abdullah et al., 2018; Shalmani et al., 2019; Sun et al., 2020). *AtZFHD5*, a ZF-HD gene in Arabidopsis, plays an important role in bud regeneration and participates in ABA and cytokinin response (Wang et al., 2011; Kim et al., 2019). Shin-Young et al. (2011) found that the leaves of Arabidopsis expressing *AtZFHD5* were larger and grew faster. Moreover, AtMIF1 may interact with a ZF-HD protein and interfere with the normal function of ZF-HD protein. Fifteen known *ZFHD* genes in upland cotton are involved in the early development and pigment synthesis of fibers (Abdullah et al., 2018). Overexpression of rice *OsZFHD1* and *OsZFHD2* resulted in the back curling of leaves (Xu et al., 2014). *CoZFHD16* participates in the regulation of coconut fruit growth and development (Sun et al., 2020).

Arabidopsis *AtZFHD1* can respond to salt, ABA and drought treatment, and can specifically bind to the ERD1 promoter (Tran et al., 2007; Wang et al., 2014). Most of the ZF-HD genes in tomato are expressed in flower buds, and several of them respond to abiotic stress and hormone treatment (Khadiza et al., 2017). There are 31 ZF-HD genes in the genome of Chinese cabbage, including seven *BraMIF* genes and 24 *BraZFHD* genes. Their expression is regulated by abiotic stress, vernalization, and photoperiod induction (Wang et al., 2016). In barley, *HvZFHD1* is regulated by dehydration, salt stress and heat stress. All increased the expression of *HvZFHD1* (Abu-Romman and Al-Hadid, 2017).

Recently, *OFP* genes have been implicated in abiotic stress response in Arabidopsis, rice (Ma et al., 2017; Tang et al., 2018). To date, there have been few studies of the OFP transcription factors in fruit trees, including peach where salt stress restricts the healthy development of the peach industry. Salt stress/resistance in peach trees would greatly benefit the industrial goal of safe production, ecological cultivation, and quality improvement. This study addresses the function of the *PpOFP1* gene in peach, and explores its biological role in response to salt stress. We analyzed both protein function and gene expression in transgenic plants in attempt to provide a new genetic improvement of important characters of peach.

## Materials and Methods

### Plant Materials

In this study, the expression pattern of *PpOFP1* and *PpZFHD1* was detected in various tissues including root, stem, leaf, flower, seed, pericarp, and mesocarp of the peach cultivar “Zhongnongpan No. 10” at normal growth condition. The peach plants which have grown for 40 days after seeds germination were treated with 200 mM NaCl, and within 24 h at unequal time interval, the leaf was collected for qRT-PCR. Transgenic tomato over-expressing *PpOFP1* and wild-type which have grown for 50 days after seeds germination were treated with 150 mM NaCl, and within 21 days at every 7 days, the leaf was collected for qRT-PCR. Tissues were collected and frozen in liquid nitrogen, and stored at −80°C for later use.

### Bioinformatics Analysis

The proteome data and annotation files for peach were download from GDR^1^. The whole proteome data and annotation files of *Arabidopsis thaliana* were downloaded from phytozome v12.1 database^2^. The alignment file including 2588 ZF-HD_dimer domain protein sequences (PF04770) was downloaded from the pfam database^3^. The hmmer model was established from the alignment file and was used to search the peach and *Arabidopsis* proteome databases using Hmmer 3.1b software^4^. Candidate genes with *e* value less than 1 × 10^–10^ were selected and validated with the NCBI-CDD online tool^5^. The ZF-HD_dimer domain proteins in peach and Arabidopsis were aligned using the MUSCLE program^6^. The neighbor joining (NJ) method in Mega 7^7^ was used to construct a phylogenetic tree where a bootstrap value was set at 1,000 times. The evolutionary tree was embellished by Evolview software^8^. The orthologous gene of *PpZFHD1* in *Arabidopsis thaliana* was identified using the MCScanX package^9^ with default setting. Subcellular localization was predicted by Cell-PLoc 2.0^10^. The promoter *cis*-elements were analyzed using the online software PlantCARE^11^.

### RNA Extraction and Quantitative Real-Time Polymerase Chain Reaction

Total RNA was extracted from ∼0.3 g of each tissue with the RNAprep Pure Plant Kit (TianGen, Beijing, China) according to the manufacturer’s instructions. First-strand cDNA was synthetized with HIScript^®^ III RT SuperMix for qPCR (+gDNA wiper) (Vazyme, Nanjing, China) according to the manufacturer’s instructions. qRT-PCR was conducted using ChamQ^TM^ Universal SYBR^®^ qPCR Master Mix (Without ROX) (Vazyme, Nanjing, China) on a CFX96 real-time PCR detection system (Bio-Rad). Each assay was done in triplicate or greater. The relative expressions were estimated using the 2^–ΔΔCT^ method (Livak and Schmittgen, 2001) with the *Ppactin* and *SIactin* genes as the internal reference. The qPCR primers are listed in Supplementary Table 1.

### Production of Yeast Lines Over-Expressing *PpOFP1* and *PpZFHD1*

The full-length coding sequence (CDS) of *PpOFP1* and *PpZFHD1* were downloaded from GDR (see text footnote 1) and amplified using the leaf cDNA. The primers are listed in Supplementary Table 2. The fragment was inserted into the pYES2 vector, and the positive over-expression colonies were propagated, and the plasmids were extracted and transferred into INVSc1 yeast. The OD value of yeast solution was adjusted to 1.2 at the wavelength of 600 nm, and then diluted to 10^–1^, 10^–2^, 10^–3^, 10^–4^, and 10^–5^. They were plated on -U solid medium with NaCl concentrations of 0, 200, and 400 mM, and cultured at 30°C for 72 h for observation of growth status.

### Production of Transgenic Tomato Lines Over-Expressing *PpOFP1*

The *PpOFP1* CDS was amplified using the leaf cDNA and spliced into the pZp211-GFP to form 35S::PpOFP1-GFP construct. The primers are listed in Supplementary Table 2. The resultant construct was introduced into *Agrobacterium tumefaciens* GV3101. Tomato sections were then transformed with *Agrobacterium* and transferred to selective medium containing kanamycin. Identification of transgenic plants was performed by PCR and qRT-PCR analysis. To verify that the tomato was an over-expression line, DNA was isolated from ∼0.3 g leaf tissue by the DNAsecure Plant Kit (TianGen, Beijing, China) according to the manufacturer’s instruction. The PCR products were visualized using agarose gel electrophoresis. qRT-PCR analysis was performed as above.

### Morphological Analysis of Leaf and Stem

Microscopic observation of leaf and stem morphology were performed by paraffin sectioning according to Wang et al. (2020). For the scanning electron microscopy, the samples were treated, and micrographs were taken as described by Li et al. (2002).

### Determination of Physiological Indexes

Physiological indexes including chlorophyll content, electrical conductivity, MDA content, soluble sugar content, soluble protein content, proline content, and antioxidant enzyme (SOD, POD, and CAT) activity of plant leaves were determined according to Zhao et al. (2002). At each sampling time point, at least three biological replicates were performed for each sample (over-expressor and wild-type lines). We totally sampled four times (0, 7, 14, and 21 days) for over-expressor and wild-type lines, respectively. The Analysis of Variance (ANOVA) was performed based on comparison among the four sampling time points for over-expressor and wild-type lines, respectively.

### Subcellular Localization of PpOFP1 and PpZFHD1 Proteins

The full-length CDS of *PpOFP1* and *PpZFHD1* without stop codons were amplified and inserted into the PRI-GFP (35S::GFP) vector for detection of subcellular localization. The primers are listed in Supplementary Table 2. 35S::PpOFP1-GFP, 35S::PpZFHD1-GFP and the control 35S::GFP construct was used to infect onion epidermal cells *via Agrobacterium tumefaciens* strain GV3101. After 72 h incubation, the GFP fluorescence signals in the transformed onion cells were observed using a Zeiss LSM880 fluorescence microscope, and the images were analyzed using the ZEN lite software (Zeiss).

### Yeast Two-Hybrid Assays

The coding region of the *PpOFP1* gene and various *PpOFP1* deletion derivatives were individually cloned into the pGBKT7 vector (Clontech) as bait, and the coding region of the *PpZFHD1* gene, and various *PpZFHD1* deletion derivatives were cloned into the pGADT7 vector (Clontech) as prey. The primers are listed in Supplementary Table 2. Different combinations of bait and prey vectors were transformed into the yeast strain Y2H cell and initially selected on selective medium (SD/–Trp/–Leu) at 30°C for ∼72 h. After the yeast cells had grown, the putative transformants were transferred to selective medium SD/-Leu/-Trp/-His/-Ade and SD/–Leu/–Trp/–His/–Ade with X-α-gal.

### Bimolecular Fluorescence Complementary Assays

The coding regions of *PpOFP1* and *PpZFHD1* without stop codon were amplified using PCR with appropriate primers in Supplementary Table 1 and cloned into pSPYNE-35S and pSPYCE-35S, respectively. The primers are listed in Supplementary Table 2. The resulting constructs were transformed into the *Agrobacterium tumefaciens* LBA4404 strain. The PpOFP1-pSPYNE and PpZFHD1-pSPYCE plasmids were mixed together (1:1), after which onion epidermal cells were transfected with the mixture for 30 min at 28°C; a mixture of PpOFP1-pSPYNE and pSPYCE was used as a control. The onion epidermal cells were then transferred to solid medium. After 48 h at 28°C, the onion epidermal cells were observed at an excitation wavelength of 488 nm under a laser scanning confocal microscope (LSM880) (Carl Zeiss, Oberkochen, Germany).

### Statistical Analysis

Statistical analysis was performed with IBM SPSS Statistics 25 software, and Duncan’s test was used to analyze significance (*P* < 0.05).

## Results and Analysis

### Expression Pattern, Subcellular Localization and Transgenic Validation of *PpOFP1*

**FIGURE 1:**
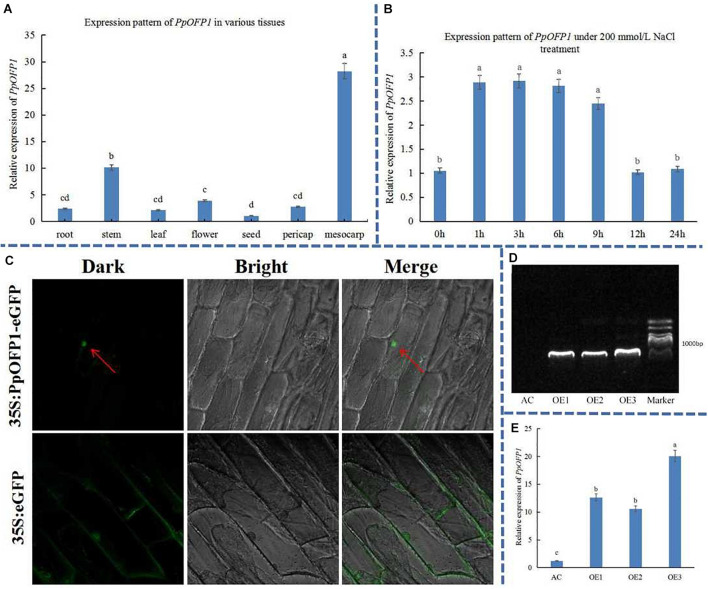
Expression pattern, subcellular localization, and transgenic validation of *PpOFP1*. **(A)** Expression pattern of *PpOFP1* in various tissues. **(B)** Expression pattern of *PpOFP1* under 200 mM NaCl treatment. **(C)** Detection of subcellular localization of PpOFP1. Red arrows indicate where a green fluorescence signal was detected in the nucleus. **(D)** Transgenic validation of *PpOFP1* by DNA electrophoresis. **(E)** Transgenic validation of *PpOFP1* by qRT-PCR. AC is wild-type, OE1, OE2, and OE3 are over-expressor lines of *PpOFP1*. DNA maker is 2,000 bp. The primer pairs used for cloning and qRT-PCR of *PpOFP1* is listed in Supplementary Tables 1, 2. Different lower-case letters indicate significant differences between means as measured by ANOVA followed by Duncan’s multiple range test (*P* < 0.05, the same below).

The expression pattern of *PpOFP1* was detected in various tissues (root, stem, leaf, flower, seed, exocarp, and mesocarp) under normal growth conditions (Figure 1A). The results showed that the *PpOFP1* was expressed in all tissues, and that higher expression was found in mesocarp and stem, followed by root and flower. The expression of *PpOFP1* in leaf was induced by ∼3 times after 1 h treatment with 200 mM NaCl, and decreased sharply after 12 h (Figure 1B), indicating that *PpOFP1* could respond to high salt stress.

The PpOFP1 protein was predicted to be located in the nucleus (Supplementary Figure 1) by Cell-PLoc 2.0 tool (see text footnote 10). To validate the subcellular localization of PpOFP1, onion epidermal cells were transfected with *Agrobacterium tumefaciens* carrying the PpOFP1-GFP construct. The green fluorescence signal of cells transfected with the 35S::PpOFP1-GFP construct was only detected in the nucleus (Figure 1C), while that of 35S::GFP empty vector was dispersed throughout the whole cell, indicating that the protein of PpOFP1 was localized in the nucleus.

Three independent transgenic tomato plants over-expressing *PpOFP1* (OE1, OE2, and OE3) were obtained. DNA electrophoresis showed that the size of bands of these *PpOFP1* over-expressors were expected (Figure 1D), and sequencing confirmed that they were *PpOFP1*. qRT-PCR results showed that the expression of *PpOFP1* was upregulated by 10–20 fold in the over-expressor lines (Figure 1E).

### Phenotypic Analysis of Transgenic Tomato Over-Expressing *PpOFP1*

**FIGURE 2:**
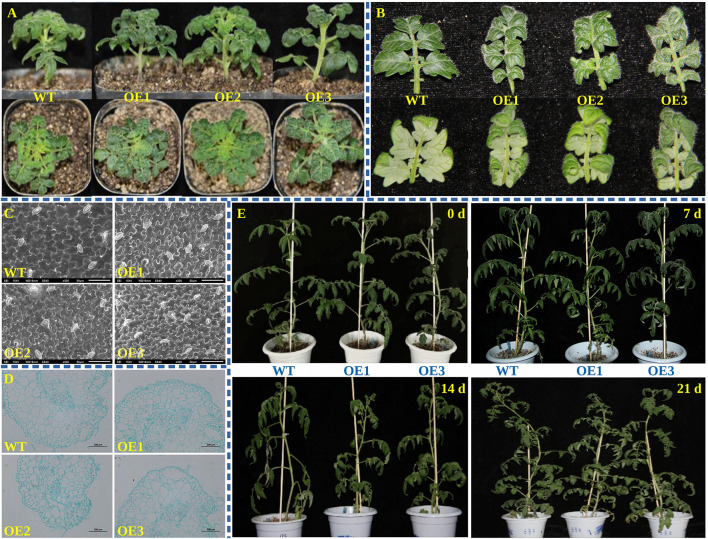
Phenotypic analysis of transgenic tomato over-expressing *PpOFP1.* **(A)** Phenotypes of whole transgenic tomato under normal growth conditions. **(B)** Leaf phenotypes of transgenic tomato under normal growth conditions. **(C)** Scanning electron microscopy of leaf epidermal cell structure under normal growth conditions. **(D)** Cross-section of paraffin embedded stem cell tissue under normal growth conditions. **(E)** Phenotype of transgenic tomato under high salt stress.

Under normal growth conditions the transgenic lines had smaller and curled leaves, thicker stems, and thinner branches than the wild-type plants (Figures 2A,B). Scanning electron microscopy (Figure 2C) bright field images (Figure 2D) show that the structure of leaf epidermal cells of transgenic lines was more compact, flatter, longer, and had a significantly increased stomata density (Figure 2C). The length and width of stem cells were reduced, the shape was irregular, and the edge was wrinkled (Figure 2D). These results suggest that *PpOFP1* may play a regulatory role in growth and development of leaf and stem.

Before salt stress (0 day), both wild-type and transgenic tomato grew vigorously (Figure 2E). After 7 days treatment with 150 mM NaCl, tomato plants appeared somewhat wilted, but the leaf curling and wilting of wild-type was more pronounced (Figure 2E). On the 14th day, the wild-type showed severe wilting and partial necrosis, and the lateral branches drooped. The growth state of transgenic tomato leaves, however, was better than that of wild-type plants, and the leaf wilting was less (Figure 2E). The tomato plants were rewatered without salt after 14 days, and both wild-type and transgenic tomato basically returned to normal status (Figure 2E). This indicated that the salt tolerance of transgenic tomato was better than that of wild-type.

### Assays of Physiological Indexes Change of Tomato Over-Expressing PpOFP1 Under Salt Stress

**FIGURE 3:**
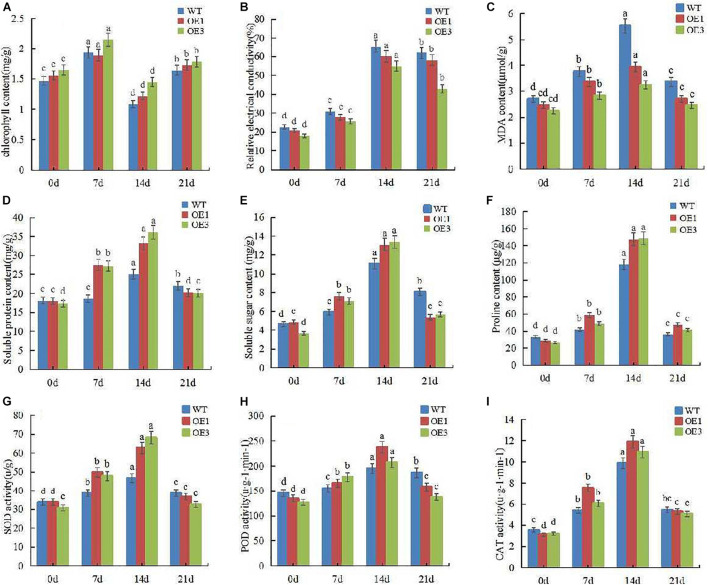
Determination of various physiological indexes of tomato leaf receiving 150 mM NaCl followed by no-salt watering after 14 days. **(A)** Chlorophyll content of leaf under 150 mM NaCl and rewatering. **(B)** Relative conductivity of leaf under 150 mM NaCl and rewatering. **(C)** MDA content of leaf under 150 mM NaCl and rewatering. **(D)** Soluble protein content of leaf under 150 mM NaCl and rewatering. **(E)** Soluble sugar content of leaf under 150 mM NaCl and rewatering. **(F)** Proline content of leaf under 150 mM NaCl and rewatering. **(G)** SOD activity of leaf under 150 mM NaCl and rewatering. **(H)** POD activity of leaf under 150 mM NaCl and rewatering. **(I)** CAT activity of leaf under 150 mM NaCl and rewatering. Different lower-case letters indicate significant differences between means as measured by ANOVA followed by Duncan’s multiple range test (*P* < 0.05, the same below).

To further explore the physiological mechanism of *PpOFP1* to salt stress, we measured a variety of physiological indexes of wild-type and transgenic tomato after salt stress. The results showed that the chlorophyll content in leaf increased at first, but then decreased under salt stress. After rehydration with normal water, the chlorophyll content returned to the initial level. Throughout whole treatment process, the chlorophyll content of wild-type plants was always lower than that of transgenic plants (Figure 3A). The relative conductivity and MDA content of leaves increased with stress time, and those values for wild-type were always higher than for transgenic plants (Figures 3B,C). The content of soluble sugar and soluble protein also increased with stress time, the accumulation in transgenic tomato was more rapid than in wild-type. After rewatering, the content of soluble sugar and soluble protein in transgenic tomato leaves decreased significantly (Figures 3D,E). The content of proline increased significantly during the 14 days of salt stress, and decreased to normal level after 7 days of rehydration. Throughout the whole salt stress, the proline accumulation of transgenic tomato was higher than that of wild-type (Figure 3F). Figures 3G–I show that the enzyme activity increased with salt stress time, and the enzyme activity of transgenic plants increased significantly. But after re-watering, the enzyme activity decreased close to the normal level, indicating that the ability to remove reactive oxygen species was higher in transgenic plants than that of wild-type. These results further confirmed that the salt tolerance of transgenic lines was significantly higher than that of wild type.

### Verification of Interaction Between PpOFP1 and PpZFHD1 Protein

**FIGURE 4:**
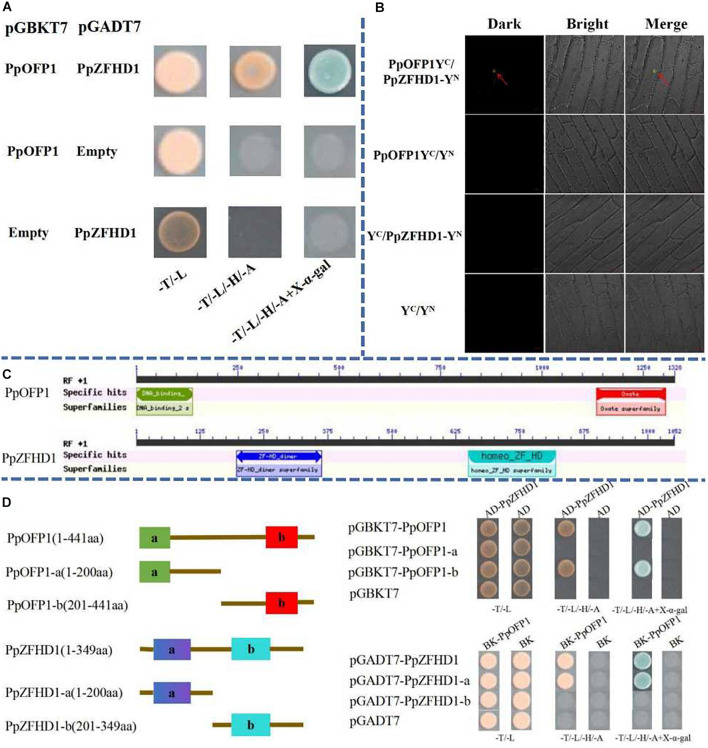
Interaction between *PpOFP1* and *PpZFHD1* proteins. **(A)** Interaction between *PpOFP1* and *PpZFHD1* proteins was verified by yeast double hybridization assay. **(B)** Interaction between *PpOFP1* and *PpZFHD1* proteins was verified by BiFC. Red arrows indicate where a green fluorescence signal was detected in the nucleus. **(C)** Conserved domains in *PpOFP1* and *PpZFHD1* proteins were predicted by the NCBI CDD online tool (https://www.ncbi.nlm.nih.gov/Structure/cdd/wrpsb.cgi). *PpOFP1* has two conserved domains: the N-terminal DNA-binding domain (PF13724) and the C-terminal OVATE domain (PF04844). There are also two domains in *PpZFHD1*, the ZF-HD_dimer domain (PF04770) at the N-terminal and the homeo_ZF_HD domain (TIGR01565) at the C-terminal. **(D)** Identification of the interaction region between *PpOFP1* and *PpZFHD1*. a and b of *PpOFP1* protein in **(D)** indicate the N-terminal DNA-binding domain (PF13724) and the C-terminal OVATE domain (PF04844), respectively. a and b of *PpZFHD1* protein in **(D)** indicate the ZF-HD_dimer domain (PF04770) at the N-terminal and the homeo_ZF_HD domain (TIGR01565) at the C-terminal, respectively.

The full-length CDS of *PpZFHD1* was amplified and ligated to the pGADT7 vector, and transformed to yeast cell line Y2H along with the PpOFP1-pGBKT7 construct. Interaction was verified using a SD/-T/-L, SD/-T/-L/-H/-A, and SD/-T/-L/-H/-A + X-α-gal plate. Figure 4A shows the pink yeast growing normally on both SD/-T/-L and SD/-T/-L/-H/-A plates, while yeast colonies on the SD/-T/-L/-H/-A + X-α-gal plate turned blue, indicating an interaction between the PpOFP1 and PpZFHD1 proteins.

To verify that PpOFP1 and PpZFHD1 can interact *in vivo*, a double molecule fluorescence complementary experiment was conducted. The results showed that the onion epidermal cells co-infected by PpZFHD1-YN and PpOFP1-YC produced the yellow fluorescence signal, while no fluorescence was seen the in other co-infections: PpZFHD1-YN and YC; YN and PpOFP1-YC; and YC and YN (Figure 4B).

To further explore which domains of PpOFP1 and PpZFHD1 interact, we conducted a domains segmentation experiment. As depicted in Figure 4C, PpOFP1 has two conserved domains: the N-terminal DNA-binding domain (PF13724) and the C-terminal OVATE domain (PF04844). There are also two domains in PpZFHD1, the ZF-HD_dimer domain (PF04770) at the N-terminal and the homeo_ZF_HD domain (TIGR01565) at the C-terminal. Different combinations of functional domains were co-transformed into Y2H yeast cells, and interactions were detected with SD/-Trp/-Leu and SD/-Trp/-His/-Trp/-Ade plates. The results show that the combination of the OVATE domain of PpOFP1 and the ZF-HD_dimer domain of PpZFHD1 can grow on SD/-Trp/-His/-Trp/-Ade plates and become blue (Figure 4D). It is suggested that the OVATE domain of PpOFP1 and ZF-HD_dimer domain of PpZFHD1 are very important in the interaction between PpOFP1 and PpZFHD1, while the N-terminal DNA-binding domain of PpOFP1 and the C-terminal homeo_ZF_HD domain of PpZFHD1 do not affect the interaction between PpOFP1 and PpZFHD1.

### Expression Pattern, Subcellular Localization and Transgenic Validation of *PpZFHD1*

We have analyzed the domain organization of ZF-HD family members, which included ZF-HD_dimer domain (PF04770). We identified 17 and 10 family members from the whole genomes of Arabidopsis and peach, respectively. Searching for protein conserved domains through NCBI-CDD online tool, the above ZF-HD proteins all contain ZF-HD_dimer domain. As the phylogenetic tree shown (Supplementary Figure 2), PpZFHD1 (Prupe.1G274700.1) closer clustered with AtZFHD1 (At1G69600.1) than other members. Collinearity analysis flanking PpZFHD1 (Prupe.1G274700.1) between Arabidopsis and peach suggested that orthologous gene of *PpZFHD1* in Arabidopsis is *AtZFHD1* (Supplementary Table 3).

**FIGURE 5:**
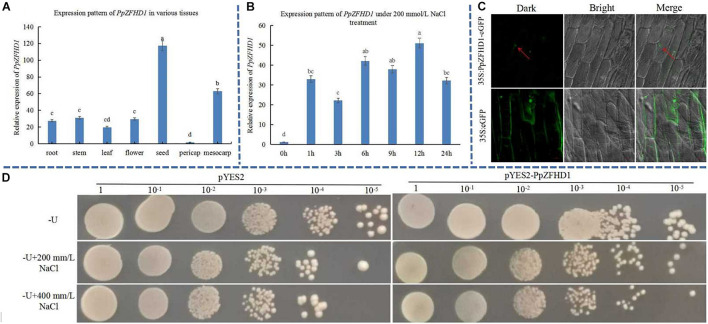
Expression pattern, subcellular localization of *PpZFHD1*, and growth status of yeast over-expressing *PpZFHD1* responsive to high salt stress. **(A)** Expression pattern of *PpZFHD1* in various tissues. **(B)** Expression pattern of *PpZFHD1* under 200 mM NaCl. **(C)** Detection of subcellular localization of PpZFHD1. **(D)** Growth status of yeast over-expressing *PpZFHD1* responsive to high salt stress. Red arrows indicate where a green fluorescence signal was detected in the nucleus. Different lower-case letters indicate significant differences between means as measured by ANOVA followed by Duncan’s multiple range test (*P* < 0.05, the same below).

The expression pattern of *PpZFHD1* was investigated in various tissues (root, stem, leaf, flower, seed, exocarp, and mesocarp) under normal growth conditions (Figure 5A). The results showed that *PpZFHD1* was expressed in all tissues, and strongest in seed and mesocarp. The results of qRT-PCR showed that after 200 mM NaCl treatment, the expression of *PpZFHD1* in leaf was strongly induced, with a peak value of 50-fold (Figure 5B), indicating that *PpZFHD1* responds to high salt stress.

The PpZFHD1 protein was predicted to be localized into the nucleus (Supplementary Figure 3) by the Cell-PLoc 2.0 tool (see text footnote 10). To accurately locate the subcellular localization of PpZFHD1, we transfected onion epidermal cells with *Agrobacterium tumefaciens* which carried the PpZFHD1-GFP construct. As shown in Figure 5C, the green fluorescence of cells transfected with 35S::PpZFHD1-GFP fusion protein fluoresced only in the nucleus, while that of cells transfected with empty vector dispersed throughout the whole cell. These results indicate that the PpZFHD1 protein was localized into the nucleus.

To further verify whether over-expression of *PpZFHD1* can confer salt tolerance to yeast, we transformed the pYES2-PpZFHD1 construct, and the empty vector pYES2 (CK) into yeast strain INVSc1. The OD value of yeast solution was adjusted to 1.2 at the wavelength of 600 nm, and then diluted to 10^–1^, 10^–2^, 10^–3^, 10^–4^, and 10^–5^. They were plated on -U solid medium with NaCl concentrations of 0, 200, and 400 mM, and cultured at 30°C for 72 h. Figure 5D indicates that the yeast plaque over-expressing *PpZFHD1* grew better than CK under high salt stress. This is consistent with that the yeast plaque over-expressing *PpOFP1* grew better than CK under high salt stress (Supplementary Figure 4).

## Discussion

### Response of PpOFP1 and PpZFHD1 to Salt Stress

If there are many stress response-related *cis*-elements in the gene promoter, the gene may participate in the responsive to abiotic stress process. In this study, we analyzed the *cis*-elements of the promoters of *PpOFP1* (Supplementary Table 4) and *PpZFHD1* (Supplementary Table 5), and found that there was a binding site (MBS) of MYB involved in drought induction in the both promoters. MYB transcription factors play a wide range of regulatory roles in stress response (Li et al., 2015). Many *ZFHD* genes have been found to be involved in abiotic stress responses. Sun et al. (2021) found that most *ZFHD* genes in tobacco could respond to drought and salt stress, and *NtZFHD21* was highly expressed in response to the drought treatments. Furthermore, gene silencing of the *NtZFHD21* gene reduced the drought resistance of tobacco. In this study, we found that the expression levels of *PpOFP1* and *PpZFHD1* were significantly increased under NaCl stress (Figures 1B, 5B). These results suggest that *PpOFP1* and *PpZFHD1* can respond to salt stress, which indicates that they may cooperate in the regulation of salt tolerance.

### PpOFP1 Promotes the Growth of Transgenic Tomato Plants

Photosynthesis is a necessary process for the growth and development of plants, which has a great impact on the yield and quality of fruit trees. Chlorophyll is the basis of plant photosynthesis, and the change of chlorophyll content is the main basis to measure plant growth. The change of chlorophyll content in this experiment indicates that stress had less damage to tomato overexpressing *PpOFP1*. We considered that the increase of stomatal density on the leaf epidermis of overexpression plants enhances photosynthesis and makes plants more resistant to adversity, and then grow more vigorously. We found that the growth of transgenic *PpOFP1* plants was significantly better than that of wild-type plants under salt stress, and the plant state recovered faster after rewatering. In this study, the leaves of transgenic tomato progenies were obviously curled and shrunk, and the arrangement of leaf epidermis cells was more compact, stomata increased significantly, which indicated that the overexpression of *PpOFP1* in tomato plants might increase drought resistance by changing leaf morphology and structure and increasing stomatal density. The salt tolerance of plants is closely related to drought tolerance (Li et al., 2019). This may be because the curled and shrunken leaves of transgenic plants reduce the water loss of leaves and increase the light area of the whole plant, so the plant grows better.

### PpOFP1 Promotes Antioxidant Enzyme Activity

Plant salt stress can damage the lipid membrane system, and malondialdehyde (MDA) is the main product of membrane lipid peroxidation. It can break many macromolecular substances that perform biological functions, such as protein, nucleic acid and enzyme (Iskender et al., 2013). Therefore, the relative conductivity and MDA content of leaves are important indicators to measure the degree of plant damage. By measuring these two indicators, we found that high salt stress caused more damage to wild-type tomato than to our transgenic plants, and that the damage degree of *PpOFP1*-OE3 was lower than that of *PpOFP1*-OE1. This may be because the expression level of *PpOFP1* in *PpOFP1*-OE3 was higher than that of *PpOFP1*-OE1, which indicated that the plants over-expressing *PpOFP1* have stronger resistance to salt stress. Further analysis of the antioxidant enzymes POD, SOD, and CAT showed that their activity of under salt stress was highest in *PpOFP1*-OE3, followed by *PpOFP1*-OE1 and finally wild type. We speculated that *PpOFP1* could protect transgenic tomato from salt stress injury by regulating the activity of antioxidant enzymes.

### *PpOFP1* Promotes the Accumulation of Osmotic Adjustment Substances

Osmotic adjustment ability is one of the most basic characteristics of plant resistance to salt, and proline is the most common osmotic regulator. Many plants accumulate proline under stress conditions. The increase of proline in stress-resistant varieties is greater than that of stress-sensitive varieties (Hayat et al., 2012). In this study, the proline content of overexpression *PpOFP1*-OE3 lines treated with salt was higher than that of wild type. In addition, soluble sugar and soluble protein also participate in osmotic regulation. Our study found that soluble sugar and soluble protein content of wild-type tomato under salt stress were lower than that of *PpOFP1* transgenic plants. *PpOFP1* may enhance salt tolerance of transgenic plants by increasing both the content of osmotic substances, and the activity of antioxidant enzymes. Overexpression of the kumquat gene *FcWRKY40* in both tobacco and lemon can promote high levels of proline and regulate ion transport, improving the tolerance of transgenic plants to salt (Dai et al., 2018). Studies with *Malus halliana* have shown that an increased accumulation of sucrose, amino acids, alkaloids, carotenoids, and other metabolites can eliminate excessive reactive oxygen species in cells and improve the salt tolerance of plants (Jia et al., 2020).

### Interaction Between PpOFP1 and PpZFHD1 Is Involved in Salt Stress

Transcription factors mostly play roles in the nucleus and regulate the normal progress of various plant reactions. The localization results of this study showed that both PpOFP1 and PpZFHD1 were located in the nucleus. This is consistent with results in other species (Liu et al., 2002; Xu et al., 2014). It indicates that both PpOFP1 and PpZFHD1 can function as transcription factors. In order to further study the regulatory network of PpOFP1 in plants, we confirmed the interaction between PpZFHD1 and PpOFP1 in the nucleus by the yeast two hybrid test and bimolecular fluorescence complementary analysis (BiFC). Moreover, the N-terminal ZF-HD_dimer domain of PpZFHD1 is rich in cysteine and histidine residues. This is the region that interacts with C-terminal OVATE domain in PpOFP1 (Figure 4D), indicating that the ZF-HD_dimer domain is an indispensable part of stable inheritance and function of ZF-HD genes. ZF-HD_dimer domains can be used as novel dimer domains to form homodimers and heterodimers, and highly conserved cysteine is essential for protein-protein interactions. Moreover, potential homodimerization and heterodimerization may enhance the transcriptional activity of the ZF-HD protein. If so, this would have a significant impact on the expression of target genes and may therefore affect the stress response (Nakashima and Yamaguchi-Shinozaki, 2010). The interaction between the ZF-HD_dimer domain of TsZFHD1 and a domain of TsNAC1 can competitively inhibit the formation of homodimer, so the co-expression of TsZFHD1 and TsNAC1 can more effectively regulate the expression of target genes (Liu et al., 2019). We speculate that this mode of action may also exist between PpZFHD1 and PpOFP1. PpOFP1 binds to ZF-HD_dimer domain through OVATE domain to inhibit the formation of self homodimer of PpZFHD1, and then more effectively regulates the expression of downstream target genes.

## Conclusion

In summary, our study demonstrates that PpOFP1 physically interacts with PpZFHD1 and confers salt tolerance to tomato and yeast. Our findings characterize the molecular mechanisms relating to PpOFP1 in peach and revealing a novel mechanism for regulating salt tolerance in peach and other perennial deciduous trees.

## Data Availability Statement

The original contributions presented in the study are included in the article/Supplementary Material, further inquiries can be directed to the corresponding author.

## Author Contributions

LL, WX, QT, and SJ designed and performed the experiments and analyzed the data. QT and SJ wrote the manuscript. All authors contributed to the article and approved the submitted version.

## Conflict of Interest

The authors declare that the research was conducted in the absence of any commercial or financial relationships that could be construed as a potential conflict of interest.

## Publisher’s Note

All claims expressed in this article are solely those of the authors and do not necessarily represent those of their affiliated organizations, or those of the publisher, the editors and the reviewers. Any product that may be evaluated in this article, or claim that may be made by its manufacturer, is not guaranteed or endorsed by the publisher.

## Footnotes

1
https://www.rosaceae.org/species/prunus_persica/genome_v2.0.a1

2
https://phytozome.jgi.doe.gov/pz/portal.html

3
http://pfam.xfam.org/family/PF04770#tabview=

4
http://hmmer.janelia.org

5
https://www.ncbi.nlm.nih.gov/Structure/cdd/wrpsb.cgi

6
http://www.drive5.com/muscle/

7
https://megasoftware.net/

8
https://www.evolgenius.info/evolview/#login

9
https://github.com/wyp1125/MCScanx

10
http://www.csbio.sjtu.edu.cn/bioinf/Cell-PLoc-2

11
http://bioinformatics.psb.ugent.be/webtools/plantcare/html/

## References

[B1] Abdullah M. Cheng X. Cao Y. P. Su X. Q. Manzoor M. A. Gao J. S. (2018). Zinc finger-homeodomain transcriptional factors (ZHDs) in upland cotton (*Gossypium hirsutum*): genome-wide identification and expression analysis in fiber development. *Front. Genet.* 9:357. 10.3389/fgene.2018.00357 30356782 PMC6189526

[B2] Abu-Romman S. Al-Hadid K. (2017). Novel zinc finger-homeodomain gene from barley (HvZFHD1) is differentially regulated during spike development and under hormonal treatments and abiotic stresses. *Not. Bot. Horti Agrobot.* 45 89–96. 10.15835/nbha45110612

[B3] Al-Shorafa W. Mahadeen A. Al-Absi K. (2014). Evaluation for salt stress tolerance in two strawberry cultivars. *Am. J. Agric. Biol. Sci.* 9 334–341. 10.3844/ajabssp.2014.334.341

[B4] Chander M. S. Palaniappan R. (2016). Oxidative stress and changes in antioxidant and biochemical constituents in papaya (*Carica papaya* L.) under salt stress. *J. Hortic. Sci.* 2 134–138.

[B5] Dai W. S. Wang M. Gong X. Q. Liu J. H. (2018). The transcription factor FcWRKY40 of *Fortunella crassifolia* functions positively in salt tolerance through modulation of ion homeostasis and proline biosynthesis by directly regulating SOS2 and P5CS1 homologs. *New Phytol.* 219 972–989. 10.1111/nph.15240 29851105

[B6] Fang S. Hou X. Liang X. (2021). Response mechanisms of plants under saline-alkali stress. *Front. Plant Sci.* 12:1049. 10.3389/fpls.2021.667458 34149764 PMC8213028

[B7] Hayat S. Hayat Q. Alyemeni M. N. Wani A. S. Pichtel J. T. Ahmad A. (2012). Role of proline under changing environments: a review. *Plant Signal. Behav.* 7 1456–1466. 10.4161/psb.21949 22951402 PMC3548871

[B8] Iskender E. Kayis T. Coskun M. Dursun O. Cogun H. Y. (2013). Changes in antioxidative enzyme activity, glycogen, lipid, protein, and malondialdehyde content in cadmium-treated *Galleria mellonella* larvae. *Ann. Entomol. Soc. Am.* 106 371–377. 10.1603/an12137 33044624

[B9] Jia X. Zhu Y. Zhang R. Zhu Z. Zhao T. Cheng L. R. (2020). Ionomic and metabolomic analyses reveal the resistance response mechanism to saline-alkali stress in *Malus halliana* seedlings. *Plant Physiol. Biochem.* 147 77–90. 10.1016/j.plaphy.2019.12.001 31846851

[B10] Khadiza K. Nath U. K. Robin A. H. K. Park J. I. Lee D. J. Kim M. B. (2017). Genome-wide analysis and expression profiling of zinc finger homeodomain (ZHD) family genes reveal likely roles in organ development and stress responses in tomato. *BMC Genomics* 18:695. 10.1186/s12864-017-4082-y 28874115 PMC5585987

[B11] Kim J. B. Kang J. Y. Park M. Y. Song M. Kim Y. C. Kim S. Y. (2019). *Arabidopsis* zinc fnger homeodomain protein ZHD5 promotes shoot regeneration and confers other cytokinin-related phenotypes when overexpressed. *Plant Cell Tissue Organ Cult.* 137 181–185. 10.1007/s11240-018-01546-7

[B12] Krishna S. S. Majumdar I. Grishin N. V. (2003). SURVEY AND SUMMARY: structural classification of zinc fingers. *Nucleic Acids Res.* 31 532–550. 10.1093/nar/gkg161 12527760 PMC140525

[B13] Li C. Ng C. K. Y. Fan L. M. (2015). MYB transcription factors, active players in abiotic stress signaling. *Environ. Exp. Bot.* 114 80–91. 10.1016/j.envexpbot.2014.06.014

[B14] Li H. Dong Q. Zhao Q. Ran K. (2019). Genome-wide identification, expression profiling, and protein-protein interaction properties of ovate family proteins in apple. *Tree Genet. Genomes* 15 1–11.30546292

[B15] Li Q. Xing L. Bai S. Lu W. Xian Z. (2002). Isolation of hag1 and its regulation by plant hormones during *in vitro* floral organogenesis in *Hyacinthus orientalis* l. *Planta* 215 533–540. 10.1007/s00425-002-0796-3 12172834

[B16] Liu F. Jiyi G. Yuke L. Yubin Z. Yin Y. (2019). Excavation of drought and salt responsive functional genes and their application in cotton breeding. *Mol. Plant Breed.* 17 7395–7400.

[B17] Liu J. H. Zhang J. Wang J. Y. Zhang J. B. Miao H. X. Jia C. H. (2017). MuMADS1 and MaOFP1 regulate fruit quality in a tomato ovate mutant. *Plant Biotechnol. J.* 16 989–1001. 10.1111/pbi.12843 28944538 PMC5902769

[B18] Liu J. van Eck J. Cong B. Tanksley S. D. (2002). A new class of regulatory genes underlying the cause of pearshaped tomato fruit. *Proc. Natl. Acad. Sci. U.S.A.* 99 13302–13306. 10.1073/pnas.162485999 12242331 PMC130628

[B19] Liu Y. Y. Douglas C. J. (2015). A role for OVATE FAMILY PROTEIN1 (OFP1) and OFP4 in a BLH6-KNAT7 multi-protein complex regulating secondary cell wall formation in *Arabidopsis thaliana*. *Plant Signal. Behav.* 10:e1033126. 10.1080/15592324.2015.1033126 26107719 PMC4622736

[B20] Livak K. J. Schmittgen T. D. (2001). Analysis of relative gene expression data using real-time quantitative pcr and the 2-ΔΔct method. *Methods* 25 402–408. 10.1006/meth.2001.1262 11846609

[B21] Ma Y. Yang C. He Y. (2017). Rice OVATE family protein 6 regulates plant development and confers resistance to drought and cold stresses. *J. Exp. Bot.* 68 4885–4898. 10.1093/jxb/erx309 29048565

[B22] Mackay J. P. Crossley M. (1998). Zinc fingers are sticking together. *Trends Biochem. Sci.* 23 1–4. 10.1016/s0968-0004(97)01168-7 9478126

[B23] Nakashima K. Yamaguchi-Shinozaki K. (2010). Regulons involved in osmotic stress-responsive and cold stress-responsive gene expression in plants. *Physiol. Plant.* 126 62–71. 10.1111/j.1399-3054.2005.00592.x

[B24] Shalmani A. Muhammad I. Sharif R. Zhao C. Ullah U. Zhang D. (2019). Zinc fnger-homeodomain genes: evolution, functional diferentiation, and expression profling under fowering-related treatments and abiotic stresses in plants. *Evol. Bioinform.* 15 1–16. 10.1177/1176934319867930 PMC6728664 31523124

[B25] Shin-Young H. Ok-Kyoung K. Sang-Gyu K. Moon-Sik Y. Chung-Mo P. (2011). Nuclear import and DNA binding of the ZHD5 transcription factor is modulated by a competitive peptide inhibitor in *Arabidopsis*. *J. Biol. Chem.* 286 1659–1668. 10.1074/jbc.m110.167692 21059647 PMC3020774

[B26] Singh A. Sharma P. C. (2018). Recent insights into physiological and molecular regulation of salt stress in fruit crops. *Adv. Plants Agric. Res.* 8 171–183.

[B27] Sivritepe N. Eriş A. (1999). Determination of salt tolerance in some grapevine cultivars (*Vitis vinifera* L.) under *in vitro* conditions. *Turk. J. Biol.* 23 473–486.

[B28] Sun J. Xie M. Li X. Li Z. Wang Q. Ding A. (2021). Systematic investigations of the ZF-HD gene family in tobacco reveal their multiple roles in abiotic stresses. *Agronomy* 11:406. 10.3390/agronomy11030406

[B29] Sun X. Fan H. Shufang G. Rui L. Fengfeng J. Yong X. (2020). Identification and bioinformatics analysis of coconut ZF-HD gene family. *Chin. J. Trop. Crops* 41:284.

[B30] Takatsuji H. (1999). Zinc-finger proteins: the classical zinc finger emerges in contemporary plant science. *Plant Mol. Biol.* 39 1073–1078. 10380795 10.1023/a:1006184519697

[B31] Tang Y. Zhang W. Yin Y. L. Feng P. Li H. Chang Y. (2018). Expression of ovate family protein 8 affects epicuticular waxes accumulation in *Arabidopsis thaliana*. *Bot. Stud.* 59 1–9. 10.1186/s40529-018-0228-8 29691677 PMC5915979

[B32] Tran L. S. P. Nakashima K. Sakuma Y. Osakabe Y. Qin F. Simpson S. D. (2007). Co-expression of the stress-inducible zinc finger homeodomain ZFHD1 and NAC transcription factors enhances expression of the ERD1 gene in *Arabidopsis*. *Plant J.* 49 46–63. 10.1111/j.1365-313x.2006.02932.x 17233795

[B33] Tsaballa A. Pasentsis K. Darzentas N. Tsaftaris A. S. (2011). Multiple evidence for the role of an Ovate-like gene in determining fruit shape in pepper. *BMC Plant Biol.* 11:46. 10.1186/1471-2229-11-46 21401913 PMC3069956

[B34] Wang H. Yin X. J. Li X. Q. Wang L. Zheng Y. Xu X. Z. (2014). Genome-wide identification, evolution and expression analysis of the grape (*Vitis vinifera* L.) zinc finger-homeodomain gene family. *Int. J. Mol. Sci.* 15 5730–5748. 10.3390/ijms15045730 24705465 PMC4013592

[B35] Wang L. Hua D. He J. Duan Y. Chen Z. Hong X. (2011). Auxin Response Factor2 (ARF2) and its regulated homeodomain gene HB33 mediate abscisic acid response in *Arabidopsis*. *PLoS Genet.* 7:e1002172. 10.1371/journal.pgen.1002172 21779177 PMC3136439

[B36] Wang Q. Xu G. Zhao X. Zhang Z. Wang X. Liu X. (2020). Transcription factor TCP20 regulates peach bud endodormancy by inhibiting DAM5/DAM6 and interacting with ABF2. *J. Exp. Bot.* 71 1585–1597. 10.1093/jxb/erz516 31740930 PMC7031059

[B37] Wang W. L. Wu P. Li Y. Hou X. L. (2016). Genome-wide analysis and expression patterns of ZF-HD transcription factors under different developmental tissues and abiotic stresses in Chinese cabbage. *Mol. Genet. Genomics* 291 1451–1464. 10.1007/s00438-015-1136-1 26546019

[B38] Windhövel A. Hein I. Dabrowa R. Stockhaus J. (2001). Characterization of a novel class of plant homeodomain proteins that bind to the C4 phosphoenolpyruvate carboxylase gene of *Flaveria trinervia*. *Plant Mol. Biol.* 45 201–214. 11289511 10.1023/a:1006450005648

[B39] Xu Y. Wang Y. Long Q. Huang J. Wang Y. Zhou K. (2014). Overexpression of OsZHD1, a zinc fnger homeodomain class homeobox transcription factor, induces abaxially curled and drooping leaf in rice. *Planta* 239 803–816. 10.1007/s00425-013-2009-7 24385091

[B40] Yang C. Shen W. He Y. Tian Z. Li J. (2016). OVATE family protein 8 positively mediates brassinosteroid signaling through interacting with the GSK3-like kinase in rice. *PLoS Genet.* 12:e1006118. 10.1371/journal.pgen.1006118 27332964 PMC4917237

[B41] Yuan Y. Y. Fang L. C. Karungo S. K. Zhang L. L. Gao Y. Y. Li S. H. (2016). Overexpression of VaPAT1, a GRAS transcription factor from *Vitis amurensis*, confers abiotic stress tolerance in *Arabidopsis*. *Plant Cell Reports* 35 655–666. 10.1007/s00299-015-1910-x 26687967

[B42] Zhang X. W. Wu J. L. Yu Q. Liu R. Y. Wang Z. Y. Sun Y. (2020). AtOFPs regulate cell elongation by modulating microtubule orientation *via* direct interaction with TONNEAU2. *Plant Science* 292 110405. 10.1016/j.plantsci.2020.110405 32005401

[B43] Zhao S. Guoan S. Xinchun D. (2002). *Experimental Guidance of Plant Physiology.* Beijing: China Agricultural Science and Technology Press.

