# Supplementary material for: OVATE Family Protein PpOFP1 Physically Interacts With PpZFHD1 and Confers Salt Tolerance to Tomato and Yeast
Source: Front Plant Sci. 2021 Nov 12;12:759955. doi: 10.3389/fpls.2021.759955 (PMC8633955; doi:10.3389/fpls.2021.759955)
Supplement: Supplementary file 1 [file Data_Sheet_1.doc]

**Supplementary data**

Table S1 Primers used for quantitative RT-PCR analysis

| Gene | Upstream primer | Downstream primer |
| --- | --- | --- |
| *PpOFP1* | ACGGGTCTCTGTCTGTCAAG | TTGGCCTGGTTGATCTTCCT |
| *PpZFHD1* | AAGGAGTGACGAGAGGTTGG | TGCCACAACCCTCATCATCT |
| *Ppactin* | CAATGCCATTCAAGCTAAGG | GAAATTCGATTTGCATGAGC |
| *SIactin* | TTTGCTGGTGATGATGCC | CCTTAGGGTTGAGAGGTGCTT |

Table S2 Primers used for various vector construct

| Gene | Upstream primer | Downstream primer |
| --- | --- | --- |
| *PpOFP1* | TCTAGAGCATGGGTAACC ACAAGTTTAG ATTATCA | GGATCCCGTTACTTGGAC CTGAGGTCAG TG |
| *PpZFHD1* | TCTAGAGCAAGGAGTGACGAGAGGTTGG | GGATCCCTGCCACAACCCTCATCATCT |
| *PpOFP1*-a | TCAGAGGACCTGCATATGATGGGTAACCACAAGTTTAGATTATCA | TCGACGGATCCCCGGGAATTCCATCCCATCAAAAGTCTCGGTAG |
| *PpOFP1*-b | TCAGAGGAGGACCTGCATATGGTTTCAATGTCGAGTTCATGCG | TCGACGGATCCCCGGGAATTCTTACTTGGACCTGAGGTCAGTGAG |
| *PpZFHD1*-a | GTACCAGATTACGCTCATATGATGGACTTTACCAGCGCTAATCA | ATGCCCACCCGGGTGGAATTCGGAAGCAAAATAGGAGGGTGG |
| *PpZFHD1*-b | GTACCAGATTACGCTCATATGCCACCCCAAATGTTGCTGG | ATGCCCACCCGGGTGGAATTCTCACGATGAAGAAGACGACCCA |


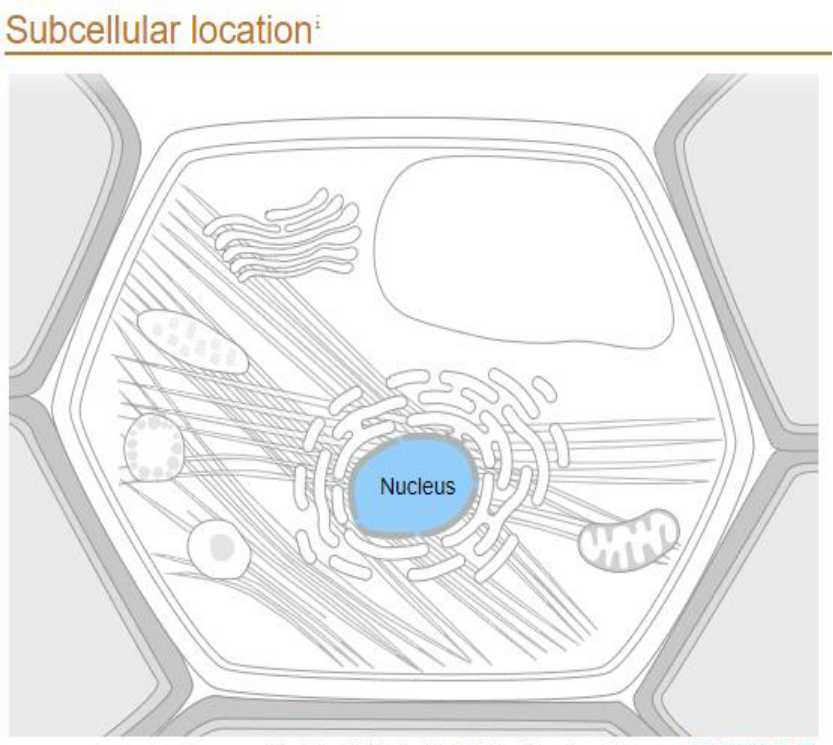


Fig. S1 Subcellular localization of PpOFP1 predicted by Cell-PLoc2.0 (http://www.csbio.sjtu.edu.cn/bioinf/Cell-PLoc-2/)


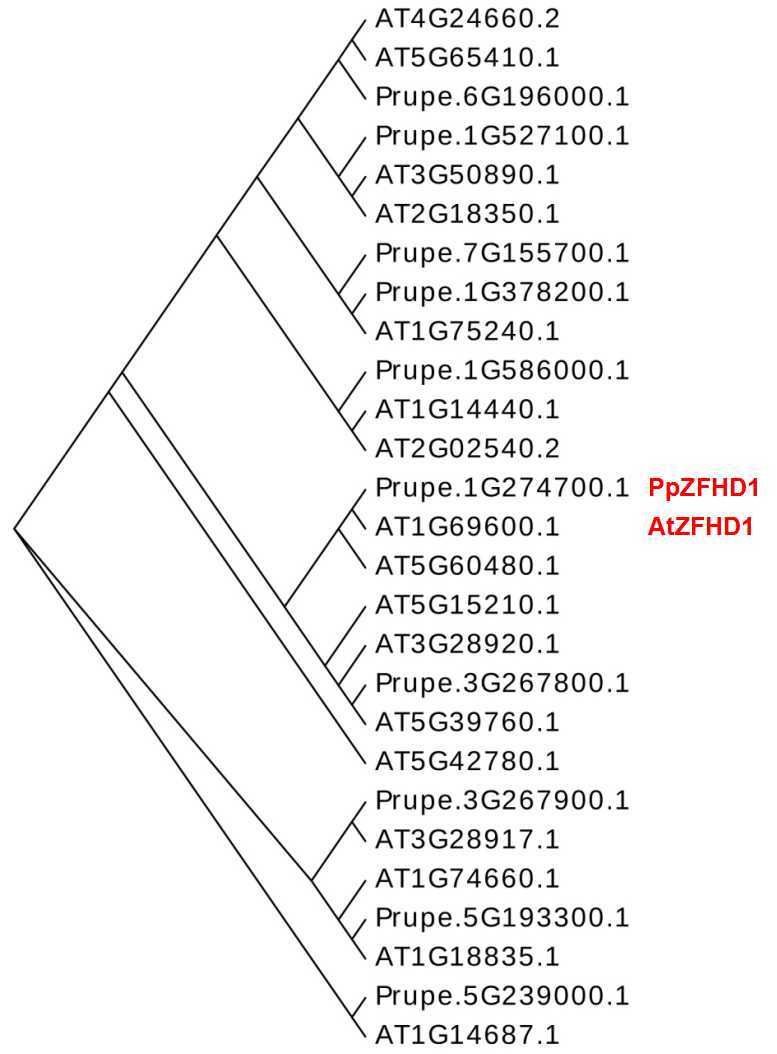


Fig. S2 Phylogenetic tree of ZF-HD family members from Arabidopsis and peach

Protein IDs beginning with ‘AT and Prupe’ are from Arabidopsis and peach genomes, respectively.

Table S3 Collinearity analysis flanking PpZFHD1 (Prupe.1G274700.1) between Arabidopsis and peach. Protein IDs beginning with ‘AT and Prupe’ are from Arabidopsis and peach genomes, respectively.

| 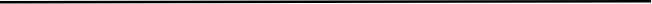N.39_of_Alignment_Block | Genes_of_AT01 | Genes_of_Pp01 | E_value_of_collinearity |
| --- | --- | --- | --- |
| 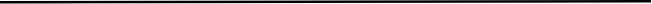 39-0 | AT1G69310.1 | Prupe.1G283500.1 | 1.00E-72 |
| 39-1 | AT1G69325.1 | Prupe.1G282700.1 | 4.00E-15 |
| 39-2 | AT1G69330.1 | Prupe.1G282600.1 | 3.00E-111 |
| 39-3 | AT1G69340.1 | Prupe.1G282100.1 | 0 |
| 39-4 | AT1G69350.1 | Prupe.1G281900.1 | 0 |
| 39-5 | AT1G69360.1 | Prupe.1G281700.1 | 2.00E-161 |
| 39-6 | AT1G69370.1 | Prupe.1G281400.1 | 1.00E-150 |
| 39-7 | AT1G69380.1 | Prupe.1G281200.1 | 1.00E-174 |
| 39-8 | AT1G69390.1 | Prupe.1G281100.1 | 7.00E-94 |
| 39-9 | AT1G69400.1 | Prupe.1G281000.1 | 2.00E-124 |
| 39-10 | AT1G69410.1 | Prupe.1G280900.1 | 2.00E-102 |
| 39-11 | AT1G69420.2 | Prupe.1G280400.1 | 0 |
| 39-12 | AT1G69430.1 | Prupe.1G280100.1 | 3.00E-151 |
| 39-13 | AT1G69440.1 | Prupe.1G279900.1 | 0 |
| 39-14 | AT1G69450.1 | Prupe.1G279800.1 | 0 |
| 39-15 | AT1G69460.1 | Prupe.1G279100.1 | 2.00E-101 |
| 39-16 | AT1G69480.1 | Prupe.1G278600.1 | 0 |
| 39-17 | AT1G69490.1 | Prupe.1G277500.1 | 3.00E-114 |
| 39-18 | AT1G69500.1 | Prupe.1G277400.1 | 0 |
| 39-19 | AT1G69510.1 | Prupe.1G277000.1 | 9.00E-39 |
| 39-20 | AT1G69520.1 | Prupe.1G276900.1 | 2.00E-77 |
| 39-21 | AT1G69530.3 | Prupe.1G276700.1 | 8.00E-154 |
| 39-22 | AT1G69540.1 | Prupe.1G276600.1 | 3.00E-98 |
| 39-23 | AT1G69560.1 | Prupe.1G276300.1 | 4.00E-75 |
| 39-24 | AT1G69570.1 | Prupe.1G276000.1 | 8.00E-58 |
| 39-25 | AT1G69580.2 | Prupe.1G275900.1 | 3.00E-86 |
| 39-26 | AT1G69588.1 | Prupe.1G274800.1 | 4.00E-11 |
| 39-27 | **AT1G69600.1** | **Prupe.1G274700.1** | 2.00E-66 |
| 39-28 | AT1G69610.1 | Prupe.1G274600.1 | 6.00E-100 |
| 39-29 | AT1G69620.1 | Prupe.1G274500.1 | 2.00E-76 |
| 39-30 | AT1G69640.1 | Prupe.1G273200.1 | 2.00E-147 |
| 39-31 | AT1G69670.1 | Prupe.1G272800.1 | 0 |
| 39-32 | AT1G69680.1 | Prupe.1G272700.1 | 1.00E-109 |
| 39-33 | AT1G69690.1 | Prupe.1G272500.1 | 3.00E-51 |
| 39-34 | AT1G69700.1 | Prupe.1G272400.1 | 3.00E-81 |
| 39-35 | AT1G69710.1 | Prupe.1G272300.1 | 0 |
| 39-36 | AT1G69760.1 | Prupe.1G272000.1 | 5.00E-27 |
| 39-37 | AT1G69780.1 | Prupe.1G271400.1 | 2.00E-119 |
| 39-38 | AT1G69790.1 | Prupe.1G270600.1 | 2.00E-102 |
| 39-39 | AT1G69800.2 | Prupe.1G269400.1 | 9.00E-169 |
| 39-40 | AT1G69810.1 | Prupe.1G269200.1 | 7.00E-63 |
| 39-41 | AT1G69830.1 | Prupe.1G268300.1 | 0 |
| 39-42 | AT1G69840.1 | Prupe.1G268100.1 | 0 |
| 39-43 | AT1G69850.1 | Prupe.1G267500.1 | 0 |
| 39-44 | AT1G69860.1 | Prupe.1G266400.1 | 0 |
| 39-45 | AT1G69890.1 | Prupe.1G265200.1 | 3.00E-106 |
| 39-46 | AT1G69910.1 | Prupe.1G265100.1 | 0 |
| 39-47 | AT1G69930.1 | Prupe.1G264700.1 | 8.00E-84 |
| 39-48 | AT1G69960.1 | Prupe.1G263000.1 | 0 |
| 39-49 | AT1G69980.1 | Prupe.1G262900.1 | 4.00E-62 |
| 39-50 | AT1G69990.1 | Prupe.1G262800.1 | 0 |
| 39-51 | AT1G70000.1 | Prupe.1G262700.1 | 5.00E-72 |
| 39-52 | AT1G70060.1 | Prupe.1G262300.1 | 0 |
| 39-53 | AT1G70070.1 | Prupe.1G262200.1 | 0 |
| 39-54 | AT1G70090.1 | Prupe.1G262000.1 | 0 |
| 39-55 | AT1G70100.3 | Prupe.1G261800.1 | 3.00E-66 |
| 39-56 | AT1G70140.1 | Prupe.1G261700.1 | 0 |
| 39-57 | AT1G70150.1 | Prupe.1G261400.1 | 2.00E-175 |
| 39-58 | AT1G70160.1 | Prupe.1G261300.1 | 0 |
| 39-59 | AT1G70170.1 | Prupe.1G261000.1 | 9.00E-144 |
| 39-60 | AT1G70180.2 | Prupe.1G260800.1 | 6.00E-33 |
| 39-61 | AT1G70200.1 | Prupe.1G259500.1 | 6.00E-57 |
| 39-62 | AT1G70210.1 | Prupe.1G259100.1 | 3.00E-135 |
| 39-63 | AT1G70230.1 | Prupe.1G258400.1 | 3.00E-174 |
| 39-64 | AT1G70260.1 | Prupe.1G257100.1 | 2.00E-72 |
| 39-65 | AT1G70270.1 | Prupe.1G256700.1 | 7.00E-11 |
| 39-66 | AT1G70280.2 | Prupe.1G256300.1 | 0 |
| 39-67 | AT1G70290.1 | Prupe.1G256200.1 | 0 |
| 39-68 | AT1G70300.1 | Prupe.1G256100.1 | 0 |
| 39-69 | AT1G70310.1 | Prupe.1G255300.1 | 0 |
| 39-70 | AT1G70330.1 | Prupe.1G254800.1 | 0 |
| 39-71 | AT1G70340.1 | Prupe.1G254000.1 | 1.00E-174 |
| 39-72 | AT1G70350.1 | Prupe.1G253900.1 | 1.00E-16 |
| 39-73 | AT1G70370.1 | Prupe.1G253700.1 | 0 |
| 39-74 | AT1G70410.2 | Prupe.1G253300.1 | 2.00E-128 |
| 39-75 | AT1G70420.1 | Prupe.1G253100.1 | 6.00E-60 |
| 39-76 | AT1G70430.1 | Prupe.1G252900.1 | 0 |
| 39-77 | AT1G70440.1 | Prupe.1G252800.1 | 3.00E-81 |
| 39-78 | AT1G70450.1 | Prupe.1G252700.1 | 2.00E-175 |
| 39-79 | AT1G70480.2 | Prupe.1G251900.1 | 2.00E-133 |
| 39-80 | AT1G70490.1 | Prupe.1G251500.1 | 2.00E-133 |
| 39-81 | AT1G70500.1 | Prupe.1G251100.1 | 0 |
| 39-82 | AT1G70505.1 | Prupe.1G250800.1 | 8.00E-81 |
| 39-83 | AT1G70510.1 | Prupe.1G249600.1 | 2.00E-124 |
| 39-84 | AT1G70520.1 | Prupe.1G249300.1 | 1.00E-160 |
| 39-85 | AT1G70540.1 | Prupe.1G249100.1 | 6.00E-15 |
| 39-86 | AT1G70550.1 | Prupe.1G248600.1 | 0 |
| 39-87 | AT1G70560.1 | Prupe.1G248200.1 | 1.00E-156 |
| 39-88 | AT1G70570.2 | Prupe.1G247400.1 | 0 |
| 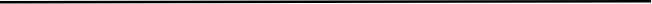 |  |  |  |


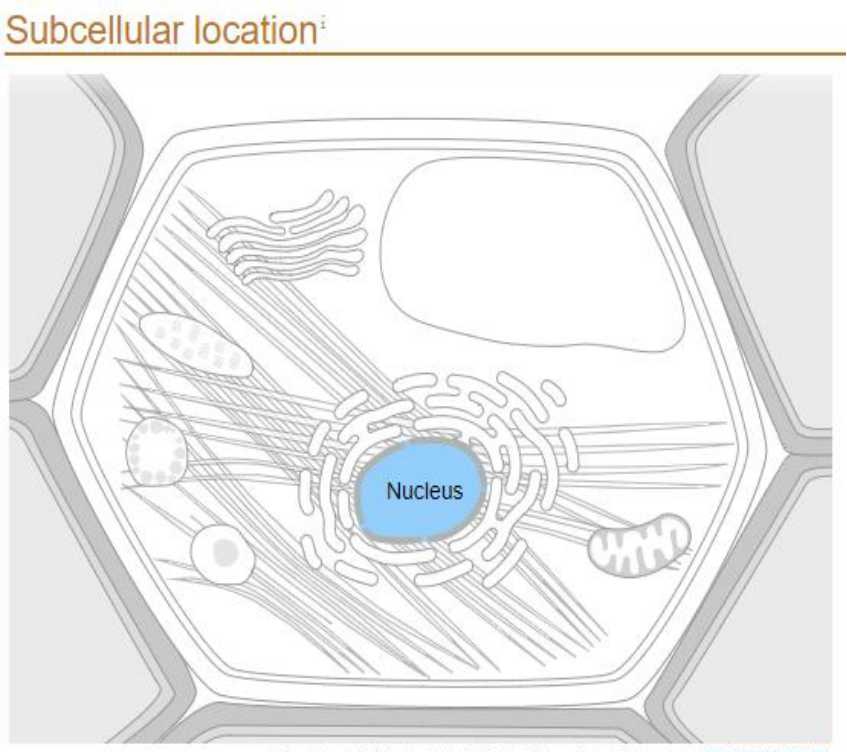


Fig. S3 Subcellular localization of PpZFHD1 predicted by Cell-PLoc2.0 (http://www.csbio.sjtu.edu.cn/bioinf/Cell-PLoc-2/)


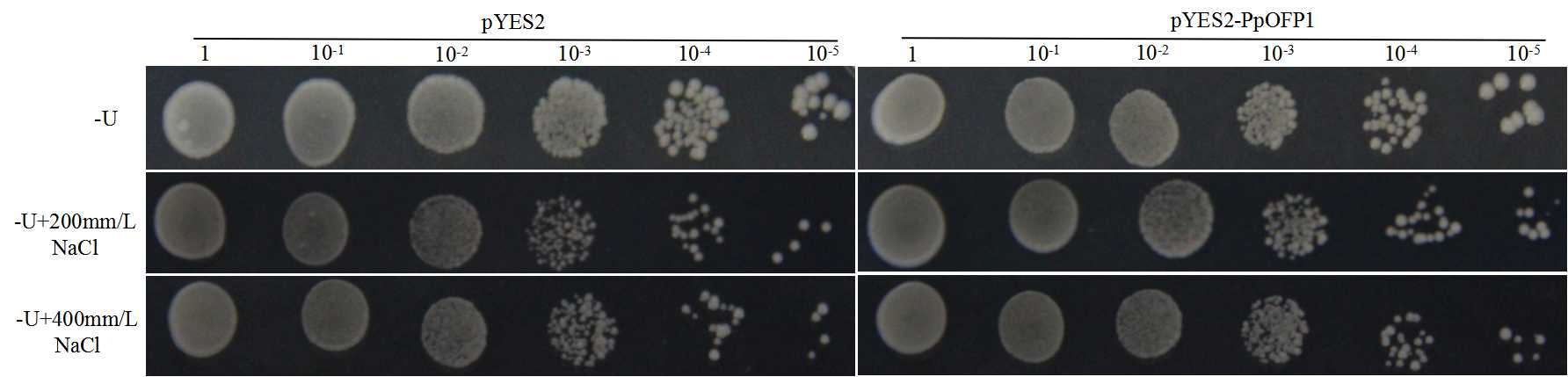


Fig. S4 Over-expression of *PpOFP1* in yeast confers the resistance of yeast to salt stress

Table S4 Promoter cis-element analysis of *PpOFP1* gene

| Function classification | Element name | Position | Motif | Element number | Functional annotation |
| --- | --- | --- | --- | --- | --- |
| Hormone signal response | ABRE | 75 | ACGTG | 1 | Abscisic acid reaction of |
|  | CGTCA-motif | 1910 | CGTCA | 1 | Methyl jasmonate reaction |
|  | P-box | 1971 | CCTTTTG | 1 | Gibberellin reaction element |
|  | TCA-element | 876,1410 1695,1967 | CCATCTTTTT | 4 | Salicylic acid reaction |
|  | TGACG-motif | 1910 | TGACG | 1 | Methyl jasmonate reaction |
| Growth and development related | ATCT-motif | 590 | AATCTAATCC | 1 | Photoreactions |
|  | CAT-box | 114 | GCCACT | 1 | Expression of meristem |
|  | G-box | 74 | TACGTG | 1 | Photoreaction |
|  | GCN4_motif | 1283,1850 | TGAGTCA | 2 | Endosperm expression |
|  | GT1-motif | 1544 | GGTTAA | 1 | Light response elements |
|  | GCN4_motif | 1283,1850 | TGAGTCA | 2 | Endosperm expression |
|  | RY-element | 504 | CATGCATG | 1 | Seed specific regulation |
|  | TCCC-motif | 1362 | TCTCCCT | 1 | Photoresponsive element |
| Response to stress | ARE | 342,847 1054,1915 | AAACCA | 4 | Anaerobic sensor |
|  | MBS | 784 | CAACTG | 1 | Myb participates in drought induced binding sites |

Table S5 Promoter cis-element analysis of *PpZFHD1* gene

| Function classification | Element name | Position | Motif | Element number | Functional annotation |
| --- | --- | --- | --- | --- | --- |
| Hormone signal response | ABRE | 781,782 1337,1339 | CACGTG,ACGTG TACGTGTC,ACGTG | 4 | Abscisic acid reaction |
|  | P-box | 1905 | CCTTTTG | 1 | Gibberellin reaction |
|  | TATC-box | 90 | TATCCCA | 1 | Gibberellin reaction |
|  | TCA-element | 881 | CCATCTTTTT | 1 | Salicylic acid reaction |
| Growth and development related | ACE | 1337 | GACACGTATG | 1 | Photoreaction |
|  | AE-box | 1136 | AGAAACAA | 1 | Light response module |
|  | ATC-motif | 578 | AGTAATCT | 1 | Photoreaction |
|  | Box 4 | 1092,1163 | ATTAAT | 1 | Photoreaction |
|  | G-box | 781,781,1339 | TACGTG | 3 | Photoreaction |
|  | GT1-motif | 1839 | GGTTAA | 1 | Light response elements |
| Response to stress | ARE | 39,552,567 947,1223,1901 | AAACCA | 6 | Anaerobic sensor |
|  | LTR | 1349 | CCGAAA | 1 | Low temperature response |
|  | MBS | 361 | CAACTG | 1 | Myb participates in drought induced binding sites |
